# Autophagy Enhancing Contributes to the Organ Protective Effect of Alpha-Lipoic Acid in Septic Rats

**DOI:** 10.3389/fimmu.2019.01491

**Published:** 2019-07-02

**Authors:** Jia Jia, Xiaoying Gong, Yang Zhao, Zhenyu Yang, Kaiqiang Ji, Ting Luan, Bin Zang, Guofu Li

**Affiliations:** Department of Critical Care Medicine, Shengjing Hospital of China Medical University, Shenyang, China

**Keywords:** sepsis, alpha-lipoic acid, autophagy, inflammation, rats

## Abstract

Alpha-lipoic acid (ALA) reportedly has protective effects against sepsis, which is a leading cause of mortality worldwide and is associated with multiple organ dysfunction. The present study aimed to investigate further the possible action mechanisms of ALA. Male Sprague-Dawley rats were subjected to cecal ligation and puncture (CLP) in order to establish a sepsis model. The rats received an oral gavage of 200 mg/kg ALA or saline immediately after surgery. The heart rate (HR), left ventricular systolic pressure (LVSP), left ventricular end-diastolic pressure (LVEDP) and maximum rising and lowering rates of left ventricular pressure (±dp/dt) were examined for assessing the cardiac function. Blood urea nitrogen (BUN) and serum creatinine levels were assessed for evaluating renal function. Neutrophil gelatinase-associated lipocalin (NAGL) was examined for reflecting acute renal injury. Histopathological alterations of the small intestine were examined by hematoxylin-eosin staining. The ultrastructure of the small intestine and kidney was observed under electron microscopy. The levels of autophagy- and inflammation-associated proteins were determined via western blot analysis. The binding of nuclear factor-kappa B (NF-κB) to DNA was tested via an electrophoretic mobility shift assay. Cell apoptosis was examined using TUNEL staining. ALA treatment improved the survival rate, restored the loss of body weight and pro-inflammatory cytokines production in the serum of CLP-induced septic rats. ALA improved the cardiac and renal functions, downregulated the expression levels of interleukin-1β, tumor necrosis factor-α, and inducible nitric oxide synthase in the myocardium and small intestine of septic rats. ALA treatment also inactivated the NF-κB signaling pathway in the small intestine. An examination of autophagy showed that ALA increased the LC3II/I ratio, upregulated Atg5, Atg7, and beclin-1 and downregulated p62 protein levels in the myocardium, kidney, and small intestine of septic rats, and further promoted autophagosome accumulation in the kidney and small intestine. In addition, ALA could also reduce cell apoptosis in myocardium, kidney and small intestine tissues. These effects can be completely or party inhibited by 3-MA. Our findings suggest that autophagy enhancing may contribute to the organ protective effect of ALA in septic rats.

## Introduction

Sepsis is a life-threatening organ dysfunction caused by the invasion of microorganisms into the bloodstream and subsequent systemic inflammatory responses. It is associated with multiple organ dysfunction (1) and is a leading cause of mortality worldwide (2, 3). Evidence shows patients who survive sepsis still suffer from long-term comorbid conditions, e.g., chronic obstructive pulmonary disease, ischemic heart disease, and malignant neoplasms, which seriously reduce quality of life and cause significant economic burdens (4, 5). Currently, rapid broad-spectrum antimicrobial therapy is recommended for the treatment of sepsis, but the heterogeneity of patients complicates clinical trials (1). Although improvements in this field have increased the survival rate of patients, sepsis remains a leading cause of death in intensive care units (6). Thus, there is still an urgent need for the development of an effective therapeutic strategy for sepsis.

Autophagy is an intracellular degradation mechanism characterized by the formation of autophagic vacuoles and the degradation of cytoplasmic content that plays a critical role in cell survival (7–9). Autophagy can be induced by cecal ligation and puncture (CLP) in septic patients and animal models (10–12). It is widely considered to be involved in organ failure and cell death in sepsis (9, 13). One prospective cohort study demonstrated that septic patients homozygous for the TT genotype of immunity-related GTPase family M, an autophagy-related polymorphic locus, suffer a significantly higher mortality rate than those carrying the CC and CT genotypes, indicating the key role of autophagy in sepsis (14). Interestingly, accumulating evidence suggests that the activation of autophagy protects against organ dysfunction (15–20). Thus, modulation of autophagy may represent a novel strategy for the treatment of sepsis.

Alpha-lipoic acid (5-[1,2-dithiolan-3-yl] pentanoic acid, ALA) is an endogenous dithiol compound that functions as a cofactor for mitochondrial enzymes. As a multifunctional antioxidant, ALA scavenges reactive oxygen species, chelates metals, and regenerates endogenous antioxidants in its oxidized and reduced forms (21, 22). ALA protects multiple organs from sepsis through its anti-inflammatory and anti-oxidative properties (23–26). In order to investigate the possible pharmacological mechanisms of ALA further, we evaluated its protective effects on small intestine, heart, and kidney via its autophagy-regulating properties in a CLP-induced rat model of sepsis.

## Materials and Methods

### Animals and Study Design

Male Sprague-Dawley rats (200–220 g) from the Experimental Animal Center of China Medical University (Shenyang, China) were maintained in a standard specific pathogen free animal room with a 12-h light/dark cycle with *ad libitum* access to water and a standard rodent diet. The rats were acclimated to the environment for 1 week prior to experimentation. All protocols were according to the Guide for Laboratory Animal Care and Use and approved by the Institutional Animal Care and Use Committee of China Medical University (IACUC No.: 2016PS051K).

The entire study was done in following experimental blocks: I: organ functions, serum proinflammatory cytokines levels, protein expression levels of Atg5 and p62, neutrophil gelatinase-associated lipocalin (NAGL) expression and cell apoptosis were examined for the revised version; II: survival rate was assessed as an independent block; III: the time course of changes in autophagy after CLP surgery was measured as an independent block; IV: the other examinations were examined in the original version. A total of 198 rats were used in the present study. For block I and IV, 42 rats for each block were used. For block II, 50 rats were used. For block III, 64 rats were used.

### CLP Procedure

Septic rats were produced using the CLP technique (27) with some modifications. Briefly, the rats were anesthetized with an intraperitoneal injection of 50 mg/kg pentobarbital followed by a 1.5-cm midline laparotomy incision. The cecum was identified, gently exteriorized, and ligated using 3–0 silk sutures. The cecum was perforated with a 22-gauge needle in three locations and replaced in the abdomen. The abdominal musculature and skin were closed by applying 2–0 silk sutures. Sham-operated animals received only laparotomy and cecum exteriorization without ligation or perforation. Antibiotics and fluid resuscitation were not used during the study.

For the time course examination, rats in the sham and CLP groups were sacrificed at 3, 6, 12, and 24 h after CLP surgery and the myocardium, kidney, and small intestine tissues were collected.

The CLP-operated rats were divided randomly using a computer random number generator into CLP, CLP+ALA, and CLP+ALA+3-methyladenine (3-MA) groups, while the sham-operated rats were divided randomly into Sham and ALA groups (*n* = 8 for each group). Rats in the same group were maintained in the same cage. Rats in the CLP+ALA+3-MA group received 15 mg/kg 3-MA (i.p.; Aladdin Regents Co., Ltd., Shanghai, China) immediately after surgery. Rats in the ALA, CLP+ALA, and CLP+ALA+3-MA groups received 200 mg/kg ALA (i.g., 40 mg/mL dissolved in saline; Sangon Biotech Co., Ltd., Shanghai, China) at 30 min after 3-MA administration, while rats in the Sham and CLP groups received the same volume of saline. The dosage of ALA was according to the previous studies (23, 24) and our preliminary experiments. At 24 h after surgery, food intake and body weight were recorded and the rats were sacrificed by deep anesthetization with an intraperitoneal injection of 150 mg/kg pentobarbital. Two rats in the CLP group died 24 h after the surgery. The experiments were performed in our laboratory and the investigators were blinded to the treatments.

### Survival Rate

For survival rate evaluation, the rats in the above 5 groups (*n* = 10 for each group) were monitored and the mortality was recorded for up to 10 days after the operation and survivors were followed for at least 2 weeks.

### Cardiac Function Examination

Cardiac function in all experimental animals was measured 24 h after CLP operation using a Biological Signal Collection System (BL-420S, Techman software Co. Ltd., Chengdu, China). Rats were anesthetized with 50 mg/kg pentobarbital, a fluid-filled catheter was advanced through the right carotid artery into the left ventricle. Heart rate, left ventricular (LV) end diastolic pressure (LVEDP), LV systolic pressure (LVSP) was recorded through a pressure-digital converter. The maximal rate of pressure rise (+dp/dt) and the maximal rate of pressure fall (-dp/dt) were calculated using the digitized LV pressure. The animal groups were randomly numbered without indicating the actual treatment. The technician examined one animal from a group at a time and then moved to the next group till the last group (round 1), and examined the second animal from each group in the second round. This procedure was repeated till the last animal was assessed.

### Histological Examinations

Tissues of interest were collected and fixed in 10% formaldehyde at 4°C overnight. The samples were embedded in paraffin and cut into 5-μm-thick sections. For pathological examinations, intestinal tissues were stained with hematoxylin (Solarbio, Beijing, China) and eosin (Sangon) (H&E). For immunohistochemical staining, kidney tissue sections were incubated with an anti-NGAL antibody (1:200; A3176; ABclonal Biotechnology Co., Ltd, Wuhan, China) at 4°C overnight and with Cy3-conjugated goat anti-rabbit secondary antibody (1:200; Beyotime Institute of Biotechnology, Haimen, China) at 37°C for 60 min. The sections were counterstained with 4′,6-diamidino-2-phenylindole and mounted with fluorescent mounting medium (Solarbio). The staining was observed under an optical microscope (DP73; Olympus, Tokyo, Japan). For terminal dexynucleotidyl transferase-mediated dUTP nick end labeling (TUNEL) staining, the tissue sections were stained using an *in situ* Cell Death Detection Kit (Roche Diagnostics, Mannheim, Germany) and counterstained with 4′,6-diamidino-2-phenylindole. The staining was observed under the DP73 optic microscope (Olympus).

### Transmission Electron Microscopy

Tissues were fixed in 2.5% glutaraldehyde for 3 h and treated with 2% osmium tetroxide for 1 h. After staining with uranyl acetate overnight, the tissues were embedded and examined under an H-7650 transmission electron microscopy (TEM) device (Hitachi High-Technologies Corp., Tokyo, Japan) at 10,000× magnification.

### Blood Urea Nitrogen (BUN), Serum Creatinine, and Nitric Oxide Assay

BUN and serum creatinine levels and nitric oxide (NO) content in small intestine tissue were assessed using BUN Assay kit, creatinine Assay kit, and NO Assay Kit purchased from Nanjing Jiancheng Bioengineering Institute (Nanjing, China) according to the manufacturer's protocol. Protein concentration was determined using a Bicinchoninic Acid (BCA) Protein Assay Kit (Beyotime).

### ELISA

Serum levels of tumor necrosis factor (TNF)-α, interleukin (IL)-1β, and IL-6 were assessed using commercial ELISA kits (MultiSciences Biotech Co. Ltd., Hangzhou, China) according to the manuscript's protocols.

### Protein Extraction and Western Blot Analysis

Whole proteins were extracted from tissues using a radio-immunoprecipitation assay lysis buffer supplemented with phenylmethanesulfonyl fluoride (Beyotime) on ice, and protein concentration was determined using a BCA Kit. Nuclear proteins were isolated using a nuclear and cytoplasmic protein extraction kit (Beyotime) following the manufacturer's instruction. Equal amounts of proteins were loaded on a sodium dodecyl sulfate polyacrylamide gel, separated by electrophoresis, and transferred to a polyvinylidene difluoride membrane (Millipore, Billerica, MA). Membranes were blocked in 5% non-fat milk and incubated with primary antibodies at 4°C overnight. The membranes were washed three times using Tris-buffered saline containing 0.1% Tween-20 and incubated with peroxidase-conjugated goat anti-mouse or goat anti-rabbit secondary antibodies (1:5,000; Beyotime). The protein blots were visualized using a chemiluminescence reagent (Beyotime). The gray values of the blots were analyzed on Gel-Pro-Analyzer with β-actin (for whole proteins and cytosolic proteins) or histone H3 (for nuclear proteins) as the internal control. The following antibodies were used (all diluted 1:1,000 unless otherwise noted): anti-LC3 (18725-1-AP), anti-TNF-α (60291-1-Ig) (Proteintech, Wuhan, China); anti-Atg7 (8558), anti-beclin-1 (3495), anti-p-p65^ser536^ (3033), anti-p65 (8242) (Cell Signaling Technology, Danvers, MA); anti-interleukin (IL)-1β (bs-0812R), anti-inhibitors of NF-κBα (IκBα) (bs-1287R) (Bioss, Beijing, China); anti-inducible nitric oxide synthase (iNOS) (1:800; ab3523; Abcam, Cambridge, UK), anti-Atg5 (1:500, A0203, ABclonal Biotechnology Co.,Ltd, Wuhan, China), anti-p62 (1: 300, BA2849, Boster Biological Technology, Wuhan, China), anti-histone H3 (KG22428) and anti β-actin (KGAA001) (both 1:500; KeyGen Biotech. Co., Ltd., Nanjing, China).

### Electrophoretic Mobility Shift Assay

The activity of nuclear factor-kappa B (NF-κB)-DNA binding in intestinal tissues was determined using a non-radioactive NF-κB electrophoretic mobility shift assay kit (Viagene Biotech, Inc., Changzhou, China) following the manufacturer's instructions.

### Statistical Analysis

The survival rate was analyzed using Kaplan-Meier log-rank test. Other results were statistically analyzed using two-way analysis of variance followed by a *post hoc* Tukey test on SPSS 19.0 (SPSS Inc., Chicago, IL). *P* < 0.05 was considered to be statistically significant. Results are presented as mean ± standard deviation (SD).

## Results

### ALA Promotes Survival Rate and Controls Inflammatory Responses in Septic Rats

**Figure 1:**
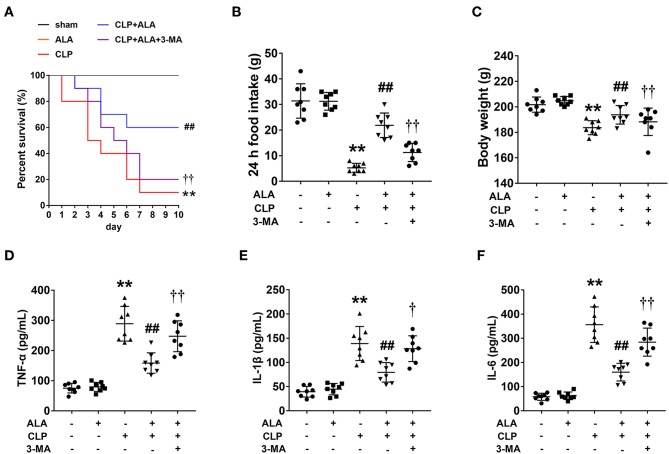
Effects of ALA on survival rate, food intake, body weight and serum pro-inflammatory cytokines in CLP-induced septic rats. ALA treatment improved the survival rate **(A)**, restored the changes in food intake **(B)**, and body weight **(C)** induced by sepsis, while 3-MA inhibited the effect of ALA. No rat in the sham or ALA groups died within 10 days, thus the survival curves of these two groups coincide with each other. ALA treatment decreased serum levels of TNF-α **(D)**, IL-1β **(E)**, and IL-6 **(F)** in CLP rats. 3-MA inhibited the effects of ALA. Data are expressed as the mean ± SD, *n* = 8. **P < 0.01 vs. sham-operated rats, ^##^*P* < 0.01 vs. CLP-operated rats, ^†^*P* < 0.05 vs. ALA-treated CLP rats, ^††^*P* < 0.01 vs. ALA-treated CLP rats.

Only 1 rat in the CLP group survived to 10 days, compared with 10 and 6 rats in the sham and CLP+ALA group, respectively. All rats in the ALA group survived for 10 days. As shown in Figure 1A, compared with the CLP rats, ALA treatment significantly increased the 10-day survival rate in CLP rats. Interestingly, 3-MA, an autophagy inhibitor, partly, but significantly eliminated this effect of ALA, which indicated that the autophagy regulation may contribute to the protective effect of ALA in septic rats.

CLP dramatically reduced food intake and caused significant body weight loss in the rats at 24 h after surgery (*P* < 0.01 vs. sham-operated rats, Figures 1B,C). These changes were restored by ALA treatment. The system inflammatory responses were evaluated by serum pro-inflammatory cytokines. As illustrated in Figures 1D–F, CLP induced markedly increased TNF-α, IL-1β and IL-6 levels in serum of CLP rats, while ALA treatment reduced the levels of these cytokines in serum, and 3-MA partly reversed this effect of ALA.

### CLP Promotes Autophagy in Septic Rats

**Figure 2:**
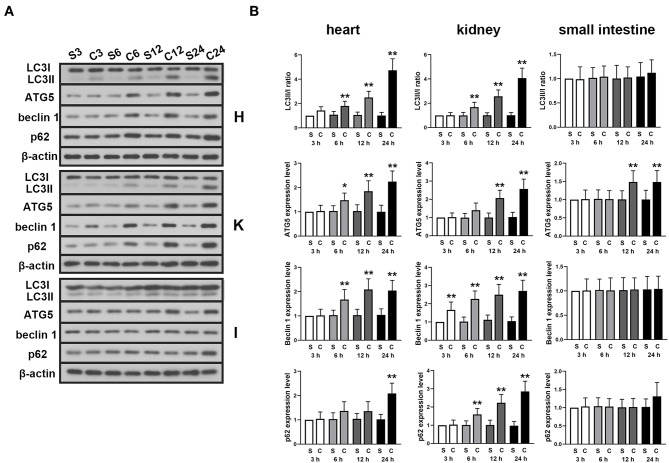
Effects of ALA on autophagy in the heart, kidney and small intestine of CLP-induced septic rats. CLP induced significantly increased LC3II/I ratio, Atg5, beclin 1 and p62 expression levels in myocardium and kidney tissues. Except Atg5, no significant change was found in these autophagy-associated proteins in small intestine tissue. **(A)** Representative protein blots of LC3, Atg5, beclin 1, and p62 in myocardium, kidney and small intestine tissues detected in 3, 6, 12, and 24 h after the CLP surgery. **(B)** Quantitative analysis of the gray values of protein blots. Data are expressed as the mean ± SD, *n* = 8. *P < 0.05 vs. sham-operated rats in the same time point, **P < 0.01 vs. sham-operated rats in the same time point. S, sham; C, CLP; H, heart; K, kidney; I, small intestine.

The expression levels of autophagy-associated proteins were detected in myocardium, kidney and small intestine tissue at different time point after the CLP surgery. The results showed that in the myocardium and kidney tissue, the LC3II/I ratio, expression levels of Atg5, beclin 1, and p62 were significantly increased with time (Figures 2A,B). However, except ATG5, the expressions of these proteins were found unchanged in small intestine.

### ALA Inhibits Inflammation and Promotes Autophagy in the Myocardium of Septic Rats

**Figure 3:**
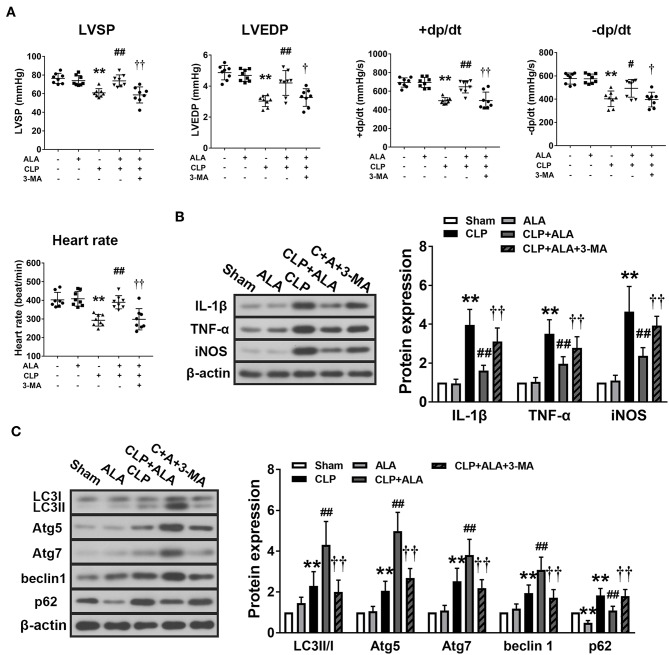
Effects of ALA on cardiac function, inflammation and autophagy in CLP-induced septic rats. **(A)** ALA elevated LVSP, LVEDP, +dp/dt, –dp/dt, and heart rate in septic rats. **(B)** ALA inhibited the expression of the pro-inflammatory factors IL-1β, TNF-α, and iNOS, and **(C)** and increased the expression of the autophagy-associated proteins LC3, Atg5, Atg7, beclin-1, and decreased the expression of p62, as determined by western blot analysis. 3-MA reversed the effects of ALA on cardiac function and these protein expressions. Data are expressed as the mean ± SD, *n* = 8. **P < 0.01 vs. sham-operated rats, ^#^*P* < 0.05 vs. CLP-operated rats, ^##^*P* < 0.01 vs. CLP-operated rats, ^†^*P* < 0.05 vs. ALA-treated CLP rats, ^††^*P* < 0.01 vs. ALA-treated CLP rats.

Cardiac function of the CLP rats was examined to evaluate the cardio-protective effect of ALA in septic rats. The results showed that CLP induced significantly decreases in heart rate, LVSP, LVEDP, and ± dt/dp, which indicated dysfunction in CLP-induced septic rats. After ALA treatment, all these parameters were markedly improved, and 3-MA eliminated this effect (Figure 3A).

At 24 h after CLP surgery, the protein levels of IL-1β, TNF-α, and iNOS were significantly increased in the myocardium compared with the sham-operated rats. Treatment with ALA restored these changes (*P* < 0.05 vs. CLP rats, Figure 3B).

In addition, the expression levels of autophagy-associated proteins in the myocardium were determined. CLP surgery significantly enhanced the LC3II/I ratio, Atg5, Atg7, and beclin-1 levels (Figure 3C). For p62, a predictor of autophagic flux (28, 29), CLP induced significant upregulation of p62, which indicated the autophagy was activated. Interestingly, ALA further increased LC3II/I ratio, Atg5, Atg7, and beclin-1 CLP rats, but not in sham-operated rats. However, ALA downregulated p62 protein expression in the sham rats. 3-MA inhibited both the anti-inflammatory and pro-autophagy effects of ALA, indicating that the anti-inflammatory effect of ALA may be associated with autophagic modulation.

### Effects of ALA on Renal Function and Autophagy in the Kidney of Septic Rats

**Figure 4:**
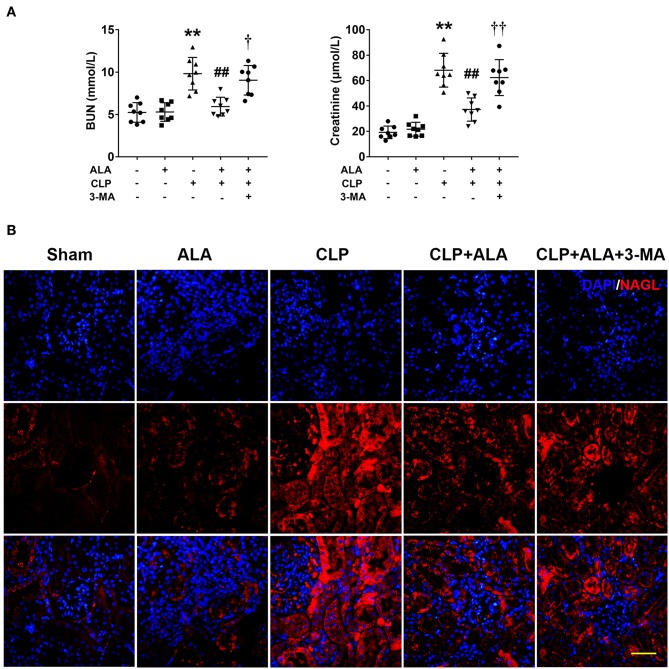
Effects of ALA on renal function of septic rats. ALA treatment reduced BUN and serum creatinine **(A)** and renal NAGL expression **(B)** in septic rats. 3-MA reversed these effects of ALA in septic rats. Scale bar: 50 μm. *n* = 8. **P < 0.01 vs. sham-operated rats; ^##^*P* < 0.01 vs. CLP-operated rats, ^†^*P* < 0.05 vs. ALA-treated CLP rats, ^††^*P* < 0.01 vs. ALA-treated CLP rats.

Renal function was evaluated by BUN and serum creatinine levels. As shown in Figure 4A, CLP operation caused kidney injury in rats as revealed by significantly increased BUN and serum creatinine levels. While ALA treated effectively reduced the levels of BUN and creatinine, and 3-MA inhibited the renoprotective effect of ALA in CLP rats. In addition, the expression of NAGL, a biomarker for acute kidney injury, was detected using immunofluorescence staining. Figure 4B showed that NAGL in the kidney was markedly upregulated after CLP surgery. ALA treatment decreased the expression of NAGL, while 3-MA partly reversed the NAGL expression.

**Figure 5:**
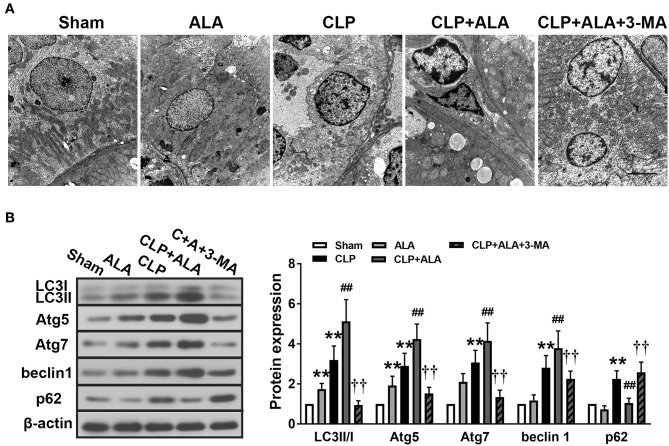
Effects of ALA on autophagy in the kidney of CLP-induced septic rats. **(A)** TEM of kidney tissue from septic rats. Scale bar: 1 μm. **(B)** Western blot analysis of LC3, Atg5, Atg7, beclin-1, and p62 expression in the kidney. ALA downregulated p62 and upregulated the other autophagy-associated proteins in the kidney of septic rats. 3-MA significantly inhibited the expression of these proteins. Data are expressed as the mean ± SD, *n* = 8. **P < 0.01 vs. sham-operated rats; ^##^*P* < 0.01 vs. CLP-operated rats, ^††^*P* < 0.01 vs. ALA-treated CLP rats.

TEM showed that CLP induced the accumulation of autophagosomes in kidney tissues (Figure 5A). Autophagosomes were also found in ALA-treated CLP rats, while ALA-induced autophagy was not obvious in sham-operated rats under TEM. Consistent with the TEM observations, CLP significantly increased the LC3II/I ratio and upregulated Atg5, Atg7, beclin-1, and p62 protein levels (Figure 5B). Similar with the observations in the intestinal and cardiac tissues, ALA treatment further increased the LC3II/I ratio and Atg5, Atg7, and beclin-1 but decreased p62 expression levels in the kidney (*P* < 0.01 vs. CLP rats), while ALA increased the LC3II/I ratio and Atg7 expression level, but not beclin-1 or p62 expression, in sham-operated rats.

### ALA Attenuates CLP-Induced Small Intestine Injury and Inflammation

**Figure 6:**
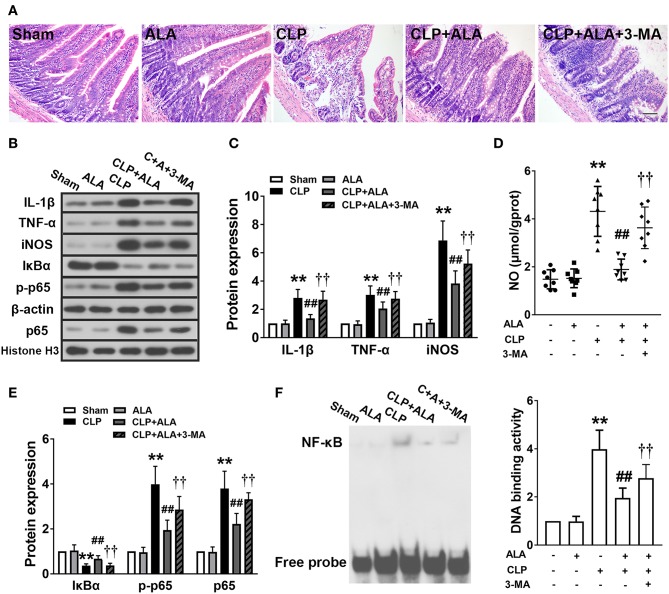
Effects of ALA on small intestine injury and inflammation in CLP-induced septic rats. **(A)** H&E staining showed that ALA treatment attenuated CLP-induced small intestine injury. Scale bar: 100 μm. **(B–E)** Western blot analysis showed that ALA treatment inhibited the production of pro-inflammatory factors **(B–D)** and activation of the NF-κB pathway **(B,E)**. Beta-actin and Histone H3 were used as housekeeper genes for whole protein and nuclear protein, respectively. **(F)** The binding activity of NF-κB to DNA was suppressed after ALA treatment. 3-MA inhibited these effects of ALA. Data are expressed as the mean ± SD, *n* = 8. **P < 0.01 vs. sham-operated rats; ^##^*P* < 0.01 vs. CLP-operated rats, ^††^*P* < 0.01 vs. ALA-treated CLP rats.

H&E staining showed that small intestine tissue exhibited an integrated structure of intestinal mucosa, well-arranged intestinal villi, and normal cell morphology. No infiltration of inflammatory cells was observed in the small intestine of sham-operated rats (Figure 6A). ALA treatment did not affect the histological structure of the small intestine, whereas the intestinal mucosa was damaged and the villi were shortened in the small intestine of the septic rats. Notable infiltration was found in necrotic mucosal epithelial cells and inflammatory cells in the mucosa. ALA administration nearly completely eliminated the presence of necrotic epithelial cells in the mucosa and decreased the infiltration of inflammatory cells. Moreover, intestinal structure was improved and the villi were arranged in an orderly manner.

The inflammatory responses in the small intestine were evaluated further by measuring the expression levels of pro-inflammatory factors. CLP significantly upregulated TNF-α and IL-1β levels (Figures 6B,C), and increased the levels of the inflammatory-associated enzyme iNOS and its product NO in the small intestine (Figures 6C,D). In addition, the activation of the typical inflammation-associated NF-κB signaling pathway was assessed. In the septic rats, IκBα expression was downregulated and the nuclear expression of NF-κB p-p65 and p65 was significantly upregulated in the small intestine (Figures 6B,E). Consistently, the activity of NF-κB-DNA binding was also enhanced in the septic rats (Figure 6F). All these changes were significantly, although not completely, reversed in ALA-treated septic rats, but not in sham-operated rats.

### ALA Promotes Autophagy in the Small Intestine of Septic Rats

**Figure 7:**
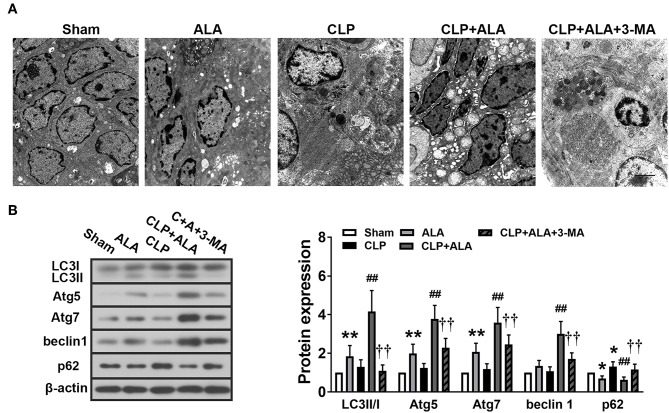
Effects of ALA on autophagy in the small intestine of CLP-induced septic rats. **(A)** The ultrastructure of the small intestine of CLP rats was observed by TEM. Scale bar: 1 μm. **(B)** Western blot analysis of LC3, Atg5, Atg7, beclin-1, and p62. ALA further activated autophagy in the small intestine of CLP rats. 3-MA inhibited these effects of ALA on autophagy in the small intestine. Data are expressed as the mean ± SD, *n* = 8. *P < 0.05 vs. sham-operated rats, **P < 0.01 vs. sham-operated rats, ^##^*P* < 0.01 vs. CLP-operated rats, ^††^*P* < 0.01 vs. ALA-treated CLP rats.

Notable autophagosomes were found in the small intestine of ALA-treated CLP rats, but not in the other groups (Figure 7A). Western blot analysis revealed that the LC3II/I ratio, Atg5, and Atg7 protein level were markedly increased and p62 protein level was decreased in the small intestine of ALA-treated sham rats, but beclin-1 expression did not change significantly (Figure 7B). In CLP-operated rats, the surgery only increased p62 protein levels, but ALA administration dramatically enhanced the LC3II/I ratio, Atg5, Atg7, and beclin-1 and reduced p62 levels. 3-MA completely or partly reversed these changes.

### ALA Inhibits Apoptosis in the Heart, Kidney, and Small Intestine

**Figure 8:**
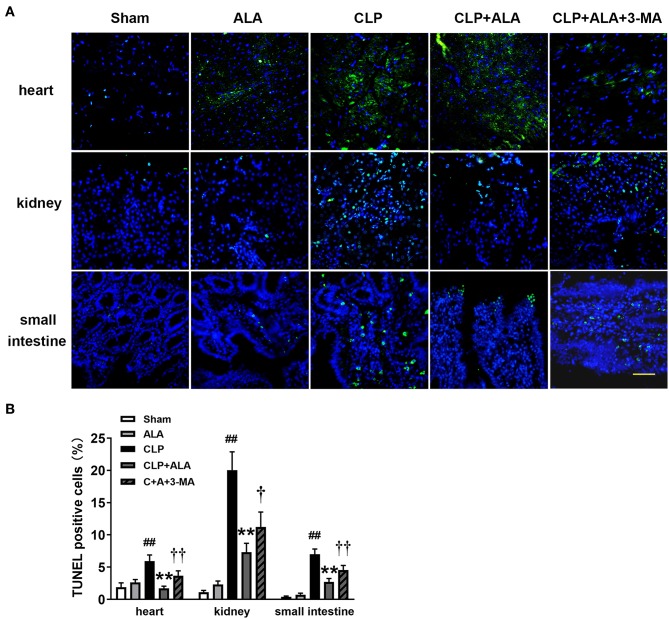
Effects of ALA on apoptosis in the heart, kidney and small intestine kidney of CLP-induced septic rats. CLP induced markedly increased TUNEL-positive cells in heart, kidney and small intestine. ALA reduced the apoptotic cells in these organs, 3-MA partly reversed this effect of ALA. **(A)** TUNEL staining of the three organs. **(B)** Quantitative analysis of the percentage of TUNEL-positive cells. Scale bar: 50 μm. Data are expressed as the mean ± SD, *n* = 8. **P < 0.01 vs. sham-operated rats; ^##^*P* < 0.01 vs. CLP-operated rats, ^†^*P* < 0.05 vs. ALA-treated CLP rats, ^††^*P* < 0.01 vs. ALA-treated CLP rats.

As shown in Figure 8, CLP surgery induced significantly increased TUNEL-positive cells in the heart, kidney, and small intestine in rats. ALA treatment has not markedly changed the percentage of TUNEL-positive cells in these tissues sham rats, but significantly reduced the percentage of TUNEL-positive cells in ALA-treated CLP rats. Interestingly, 3-MA partly but significantly restored the ALA-induced reduction of TUNEL-positive cells.

## Discussion

ALA treatment improved the survival rate of sepsis rats. In addition, reduced food intake and body weight and increased systemic inflammatory responses were also restored by ALA. Meanwhile, heart and kidney functions damaged by sepsis were improved after treatment of ALA. Local inflammatory responses in the small intestine and myocardium were inhibited, and autophagy was activated in the small intestine, myocardium, and kidney of the septic rats. All these effects were inhibited by the autophagy inhibitor 3-MA. These findings confirmed the protective functions of ALA on multiple organs in sepsis.

Sepsis leads to profound cardiac and renal dysfunction, which increases the mortality rate in sepsis (30–33). Approximately 40–50% of patients with sepsis experience myocardial depression of varying severity and 30–60% suffer from renal insufficiency (32–35). Autophagy protects multiple organs against sepsis, including the heart and kidney (36, 37). During autophagy, redundant proteins or damaged organelles are transported to lysosomes for recycling. Autophagy is activated under conditions of stress, such as inflammation or ATP depletion, to maintain cellular homeostasis (38, 39). During the initiation of autophagy, Atg12 is activated by Atg7, transferred to Atg10 and conjugated to Atg5 to form an autophagosomal precursor (40). LC3I is also activated by Atg7 and conjugated to phosphatidylethanolamine to form LC3II, which is localized to autophagosomal membranes (40). Beclin-1 plays a critical role in facilitating autophagosome maturity by interacting with the class III-type phosphoinositide 3-kinase Vps34 (41). p62 was a selected autophagy adaptor in mammals and activating autophagy reduces the expression of p62, silencing p62 can also activate autophagy (28, 42). We found that autophagy was activated in the myocardium and kidney, as evidenced by the enhancement of the LC3II/I ratio, Atg 5, Atg7, and beclin-1 expression, reduced p62 expression, and the number of autophagosomes. The activation of autophagy in the myocardium and kidney of septic rats may reflect the initiation of a self-protective mechanism. However, this compensation may be not effective enough to prevent injury completely. Impaired cardiac and renal functions were found in septic rats. ALA reportedly protects multiple organs in sepsis through its antioxidant and anti-inflammatory effects (24, 43). We found that ALA improved cardiac and renal function, further enhanced autophagy in the myocardium and kidney, and inhibited the inflammatory response in the myocardium. These findings indicate that the activation of autophagy may contribute to the cardio- and renal-protective functions of ALA in sepsis treatment.

The pharmacological activation of autophagy can attenuate sepsis-induced small intestine epithelial injury (44). Here, the histological structure and inflammatory response were impaired in the small intestine of septic rats. An assessment of autophagy showed that the expression of autophagy-associated proteins was unchanged in the small intestine of septic rats, which is consistent with a study showing that these markers were preserved at 20 h after CLP surgery (44). In addition, we found that NF-κB signaling was activated after CLP, which may contribute to the repression of autophagy in the small intestine (38). Treatment with ALA markedly attenuated small intestine injury, which may be associated with the improved food intake and body weight observed in the septic rats. These findings suggest that ALA can protect the small intestine against CLP-induced sepsis, which may be associated with the inhibition of inflammation and activation of autophagy.

In the present study, ALA also inhibited system inflammation and cell apoptosis in heart, kidney and small intestine. Inhibiting autophagy using 3-MA could partly reverse these effects of ALA, which indicates that autophagy participates in the regulation of inflammation and apoptosis. According to the previous reports, autophagy is essential for regulating the inflammation and apoptosis (45–48). Although the detail mechanisms still need to be revealed, several studies found the crosstalk between autophagy and these two processes. For inflammation, the activation of autophagy inhibits the production of pro-inflammatory cytokines (49, 50), and is regulated by the activation of the NF-κB pathway (12). For apoptosis, autophagy associated molecules can bind to, interact with, or be regulated by apoptotic factors (45, 51, 52). We found that the anti-inflammatory and anti-apoptotic effects of ALA in these organs were inhibited by 3-MA and thereby were dependent on the modulation of autophagy. Interestingly, neither inflammation nor apoptosis was completely restored by 3-MA, suggesting that some other mechanisms regulated these two processes, and autophagy activation was not the unique mechanism contributing to the beneficial effect of ALA on sepsis. Nevertheless, our results suggest that autophagy activation is associated with the protective effect of ALA against sepsis.

Protective effect of ALA against sepsis in animal models has been reported in previous studies (25, 26, 43), and our study further reveals the possible mechanism associated with autophagy. ALA is a strong antioxidant and the antioxidative effect contributes to most of its pharmacological functions including organ protection in sepsis (24). In contrast, the regulation of ALA on autophagy varies with different cell types and diseases (53). In the present study, we report for the first time that the pro-autophagic action of ALA contributes to its organ protective effects, which can be eliminated by autophagy inhibitor. This finding further reveals the possible pharmacological mechanism of ALA in sepsis treatment, which may provide clinical implications in sepsis therapy or adjuvant therapy.

At the same time, according to the Minimum Quality Threshold in Pre-clinical Sepsis Studies (54) there are some limitations in the present study: (1) To avoid the possible interferences due to the menstrual cycle and estrogen, we only used male animals. (2) The effect of ALA was evaluated only on severe sepsis model. (3) The ALA was treated immediately after CLP surgery, not post-treatment. (4) Our results were observed in a severe sepsis model established on adult health rats, the effect and mechanisms of ALA have not been investigated in moderate sepsis model or other ages. In addition, the ratio of LC3II/I was tested using whole tissue instead of cytosolic fraction, which was not as precise as using cytosolic fraction as recommended in Guidelines for the Use and Interpretation of Assays for Monitoring Autophagy (55).

In conclusion, ALA improves the survival rate of CLP-induced septic rats and attenuates sepsis-induced dysfunction of heart and kidney. The effect that it activates autophagy in the heart, kidney, and small intestine may contribute to the multiple organ protective function of ALA during sepsis treatment. This study reveals new pharmacological mechanisms of ALA and suggests that ALA is a potential candidate agent for sepsis therapy.

## Data Availability

The raw data supporting the conclusions of this manuscript will be made available by the authors, without undue reservation, to any qualified researcher.

## Ethics Statement

This study was carried out in accordance with the recommendations of The Guideline for the Care and Use of Laboratory Animals. All protocols were approved by the Institutional Animal Care and Use Committee of China Medical University (IACUC No.: 2016PS051K).

## Author Contributions

GL designed the study. JJ, XG, YZ, ZY, KJ, TL, and BZ performed the research. JJ analyzed the data. JJ and GL wrote the paper. All authors read and approved the final version of the manuscript.

### Conflict of Interest Statement

The authors declare that the research was conducted in the absence of any commercial or financial relationships that could be construed as a potential conflict of interest.
